# Targeted muscle reinnervation in above knee amputation: surgical technique

**DOI:** 10.3171/2022.10.FOCVID2293

**Published:** 2023-01-01

**Authors:** Michael J. Boctor, Julian L. Klosowiak, Simon Moradian, Iulianna Taritsa, Gregory A. Dumanian, Jason H. Ko

**Affiliations:** Division of Plastic Surgery, Department of Surgery, Northwestern Feinberg School of Medicine, Chicago, Illinois

**Keywords:** targeted muscle reinnervation, above-the-knee amputation, surgical technique

## Abstract

In the United States, an estimated 185,000 individuals undergo amputation of their upper or lower limb. This results in residual limb pain in up to 85% of cases. Targeted muscle reinnervation (TMR) is a technique that has been shown to prevent symptomatic neuroma formation. In this video, the authors demonstrate their technique utilizing TMR at the time of above-the-knee amputation. Coaptations are made to provide motor targets for branches of the saphenous, tibial, and peroneal sensory nerves. At the featured patient’s most recent follow-up visit 3 months postoperatively, she reported no stump pain or phantom limb pain.

The video can be found here: https://stream.cadmore.media/r10.3171/2022.10.FOCVID2293

## Transcript

In the United States, an estimated 185,000 people undergo amputation of their upper or lower limb.^1^ This results in residual limb pain in up to 85% of cases.^2^ Targeted muscle reinnervation is a technique that has been shown to prevent symptomatic neuroma formation.^2^ In this video, we demonstrate our technique utilizing TMR at the time of above-the-knee amputation.

### 0:53 Incision: First TMR Site.

First, we identify the ASIS and medial knee. Running between these two landmarks is the sartorius muscle, and deep to the sartorius is the saphenous nerve. We make an approximately 15-cm incision medial to the sartorius muscle. Skin and soft tissue are divided until we expose and identify the great saphenous vein. The vein is retracted medially, and sartorius fascia is incised.^3^

### 1:35 Fist Saphenous Nerve Branch.

Here you can see a saphenous nerve branch. The sartorius muscle is freed both medially and laterally. The superficial femoral artery is identified, with nearby nerves laterally.

### 2:08 First Nerve Stimulator.

We use a nerve stimulator to determine whether a nerve is sensory or motor. Upon stimulating the medial nerve, there is no contraction, suggesting it is a pure sensory branch of the saphenous nerve. When we stimulate the lateral nerve, we observe a clear muscle contraction of the sartorius muscle, indicating it is a motor nerve to the sartorius. It is important to transect the sensory nerve distally, and the motor nerve proximally, in order to maximize available nerve length and bring the nerves together under minimal tension.^4^ Our coaptation is made with two to three interrupted sutures in the epineurium using a 6-0 Prolene.

### 2:53 First Coaptation.

Here we see our result: a sensory branch of the saphenous branch connected to a motor nerve to the sartorius under a tension-free coaptation.

### 3:06 Second Nerve Stimulator.

More proximally, we once again use our nerve stimulator to identify a sensory saphenous branch over the superficial aspect of the sartorius muscle, as well as an adjacent motor branch. There was an additional nearby motor branch entering the sartorius more proximally. That motor branch is cut proximally. Then the sensory branch is cut distally, to provide maximal length. Then the first motor branch we identified is cut proximally.

### 3:45 Second Coaptation.

The next step is to bring all these nerves together. We will have one sensory branch coapted to two motor branches. We coapt to two motor branches to mitigate the size mismatch between the large sensory nerve and smaller motor nerves, and to create a fertile wound bed of denervated muscle for the sensory nerves to have somewhere to go and something to do.^5^ So to summarize, we have a sensory saphenous nerve branch plugged into two motor branches to the sartorius muscle.

### 4:19 Third Coaptation.

Finally, we find additional sensory branches of the saphenous nerve and adjacent motor nerves more distally, using our nerve stimulator. Once again, the sensory nerve is cut distally, and motor nerve is cut proximally, and the two are coapted without tension using two epineurial sutures. We typically place at least two epineurial sutures at the nerve coaptation, both for security and good approximation of the two nerve ends. We also occasionally apply Tisseel to reinforce the coaptation.

### 4:45 Summary of Saphenous Nerve Coaptations.

In summary, we have identified three sensory branches of the saphenous nerve and have three sites of nerve coaptation: one distally, one in the midportion, and one proximally with a double motor nerve coaptation.

### 4:58 Closure: First TMR Site.

Our TMR incision is closed in layers with interrupted 3-0 Vicryl and 4-0 monocryl sutures. A drain is placed at each surgical site, and the transfemoral amputation site is closed with interrupted 3-0 Vicryl sutures and staples. For the second portion of the case in a transfemoral amputee, the patient is placed in a prone position to access the posterior thigh.

### 5:16 Incision: Second TMR Site.

A 12-cm incision is made in the midportion of the thigh starting at the gluteal skin crease. Skin and soft tissue are divided until we identify the posterior femoral cutaneous nerve of the thigh. Once identified, the nerve is mobilized distally. We then divide and retract the nerve for TMR later.

### 5:45 Sciatic Nerve.

We identify the sciatic nerve by palpating between the hamstring muscles and dissect it as far distally as possible through the incision. With gentle retraction, we deliver the nerve through our incision, since it has already been transected distally as part of the amputation. You can see its division into the tibial and peroneal components here.

### 6:07 Fourth Nerve Stimulator.

Using our nerve stimulator, we identified four distinct motor nerves. These included motor branches to the semitendinosus and semimembranosus muscles, a larger 3-mm branch to the biceps femoris, as well as a smaller branch to the biceps femoris. Each motor nerve is transected proximally for maximal length.

### 6:37 Fourth Coaptation.

We then split the sciatic nerve into its tibial and peroneal components. The tibial component is cut and its distal remnant is discarded. Here we see the proximal cut end of the tibial nerve, which will be coapted to two motor nerves to the semitendinosus and semimembranosus. Note how proximally the motor nerves have been identified in the posterior thigh. This is important since it ensures a larger caliber of the motor nerve, before it has arborized into the muscle more distally, and improves the size mismatch between the large tibial and peroneal nerves. Transecting the motor nerves proximally also effectively denervates the posterior thigh musculature, resulting in more target receptors for the sensory nerves to grow into.^6^

### 7:27 Fifth Coaptation.

We then cut the peroneal component of the sciatic nerve. Here you see that larger motor branch of the biceps femoris, which is coapted to the proximal cut end of the peroneal nerve.

### 7:41 Sixth Coaptation.

Finally, we bring our posterior femoral cutaneous nerve back into the field, which, if you recall, was cut and retracted earlier. That nerve is sewn directly into the biceps femoris muscle where the small motor branch enters, mitigating some of the size mismatch and ensuring the sensory nerve is adjacent to the motor nerve and denervated muscle fibers.^5,7^

### 8:02 Summary of Sciatic Nerve Coaptations.

To summarize, we have three sites of nerve coaptations: the posterior femoral cutaneous nerve to the biceps femoris, the tibial component of the sciatic nerve to two motor branches of the semitendinosis and semimembranosis, and the peroneal component of the sciatic nerve to the biceps femoris. You can pause the video here to see a summary of the coaptations on the anterior and posterior sides.

### 8:17 Closure: Final.

A drain is placed, and the incision is closed in layers with 3-0 Vicryl and 4-0 Monocryl suture. Following TMR at the time of amputation, patients are instructed to remain non-weight-bearing to the amputated extremity for 6–8 weeks. Prosthetic usage is avoided for at least 1 month postoperatively, though early shrinker or liner wear is encouraged. At this patient’s most recent follow-up visit 3 months later, she reports no stump pain and no phantom limb pain.

## Disclosures

Dr. Ko: consultant for Checkpoint Surgical Inc., Neuraptive Therapeutics Inc., KLISBio Inc., and EDGe Surgical Inc.; and member of the Scientific Advisory Board of Mesh Suture Inc. Dr. Dumanian: consultant for Checkpoint Surgical Inc.

## Author Contributions

Assistant surgeon: Klosowiak, Moradian. Editing and drafting the video and abstract: all authors. Critically revising the work: Boctor, Klosowiak, Moradian, Dumanian, Ko. Reviewed submitted version of the work: all authors. Approved the final version of the work on behalf of all authors: Boctor. Supervision: Ko.

## References

[1] Dillingham TR Pezzin LE MacKenzie EJ Limb amputation and limb deficiency: epidemiology and recent trends in the United States South Med J 2002 95 8 875 883 12190225 10.1097/00007611-200208000-00018

[2] Dumanian GA Potter BK Mioton LM Targeted muscle reinnervation treats neuroma and phantom pain in major limb amputees: a randomized clinical trial Ann Surg 2019 270 2 238 246 30371518 10.1097/SLA.0000000000003088

[3] Janes LE Fracol ME Ko JH Dumanian GA Management of unreconstructable saphenous nerve injury with targeted muscle reinnervation Plast Reconstr Surg Glob Open 2020 8 1 e2383 32095383 10.1097/GOX.0000000000002383 PMC7015600

[4] Agnew SP Schultz AE Dumanian GA Kuiken TA Targeted reinnervation in the transfemoral amputee: a preliminary study of surgical technique Plast Reconstr Surg 2012 129 1 187 194 22186509 10.1097/PRS.0b013e3182268d0d

[5] Lanier ST Jordan SW Ko JH Dumanian GA Targeted muscle reinnervation as a solution for nerve pain Plast Reconstr Surg 2020 146 5 651e 663e 10.1097/PRS.0000000000007235 33136966

[6] Souza JM Fey NP Cheesborough JE Agnew SP Hargrove LJ Dumanian GA Advances in transfemoral amputee rehabilitation: early experience with targeted muscle reinnervation Curr Surg Rep 2014 2 5 51

[7] Valerio I Schulz SA West J Westenberg RF Eberlin KR Targeted muscle reinnervation combined with a vascularized pedicled regenerative peripheral nerve interface Plast Reconstr Surg Glob Open 2020 8 3 e2689 32537346 10.1097/GOX.0000000000002689 PMC7253250

